# Virtue or Pretense? Looking behind Self-Declared Innocence in Doping

**DOI:** 10.1371/journal.pone.0010457

**Published:** 2010-05-05

**Authors:** Andrea Petróczi, Eugene V. Aidman, Iltaf Hussain, Nawed Deshmukh, Tamás Nepusz, Martina Uvacsek, Miklós Tóth, James Barker, Declan P. Naughton

**Affiliations:** 1 School of Life Sciences, Kingston University London, Kingston upon Thames, United Kingdom; 2 Department of Psychology, University of Sheffield, Sheffield, United Kingdom; 3 Land Operations Division, Defence Science and Technology Organisation, Edinburgh, Australia; 4 School of Pharmacy and Chemistry, Kingston University London, Kingston upon Thames, United Kingdom; 5 Faculty of Physical Education and Sport Sciences, Semmelweis University, Budapest, Hungary; Pennington Biomedical Research Center, United States of America

## Abstract

**Background:**

Social science studies of doping practices in sport rely predominantly on self-reports. Studies of psychoactive drug use indicate that self-reporting is characterised by under-reporting. Likewise doping practice is likely to be equally under-reported, if not more so. This calls for more sophisticated methods for such reporting and for independent, objective validation of its results. The aims of this study were: i) to contrast self-reported doping use with objective results from chemical hair analysis and ii) to investigate the influence of the discrepancy on doping attitudes, social projection, descriptive norms and perceived pressure to use doping.

**Methodology/Principal Findings:**

A doping attitudes questionnaire was developed and combined with a response latency-based implicit association test and hair sample analysis for key doping substances in 14 athletes selected from a larger sample (N = 82) to form contrast comparison groups. Results indicate that patterns of group differences in social projection, explicit attitude about and perceived pressure to use doping, vary depending on whether the user and non-user groups are defined by self-report or objectively verified through hair analysis. Thus, self-confessed users scored higher on social projection, explicit attitude to doping and perceived pressure. However, when a doping substance was detected in the hair of an athlete who denied doping use, their self-report evidenced extreme social desirability (negative attitude, low projection and low perceived pressure) and contrasted sharply with a more positive estimate of their implicit doping attitude.

**Conclusions/Significance:**

Hair analysis for performance enhancing substances has shown considerable potential in validating athletes' doping attitude estimations and admissions of use. Results not only confirm the need for improved self-report methodology for future research in socially-sensitive domains but also indicate where the improvements are likely to come from: as chemical validation remains expensive, a more realistic promise for large scale studies and online data collection efforts is held by measures of implicit social cognition.

## Introduction

The widespread use of performance enhancing drugs [1], along with advances in performance enhancements coupled with the increasing costs of continuous development of the testing methods [2] have led anti-doping strategies to turn to identifying predictors and/or barriers of doping behaviour, over and above sanctioning. The recent debate around the practicalities and moral justification of in- and out of competition testing [1], [3] has reinforced the need for preventive measures. Social science doping research has a long standing tradition in investigating social cognition (attitudes, norms, beliefs) and personality traits in a quest to find a set of characters that clearly distinguishes athletes who engage in doping practices and those who do not [4]–[10]. Based on these differences, past research has strived to establish behavioural models [11]–[16] with the ultimate aim of being able to predict doping use and to inform anti-doping programmes for potential intervention points and strategies. To date, only a few of these models have been empirically tested [13], [15], and they are exclusively based on self-declaration of behavioural intention or behaviour; and explicit assessment of attitudes, beliefs, norms and motivation.

Previously, researchers assumed that social cognitive determinants of behaviour are accessible and explicitly endorsed by individuals, hence relied exclusively on individual's self-reports when investigating thoughts and feelings that underlie human behaviour. However, over the past two decades, convincing evidence has led to suggestions that the human mind operates in dual, conscientious and unconscientious, mode [17]–[19], therefore key components of the cognitive processes influencing behaviour are partially hidden from people's awareness or under limited ability to control. Owing to this phenomenon, it has been acknowledged that self-report measures are restricted in capturing the complexity of the cognitive processes that underlie social actions, thus social psychologists have turned to incorporating implicit assessment of the relevant cognitions. This approach has particularly intrigued researchers in socially sensitive domains where it is fair to assume that socially desirable responding is likely to confound explicit assessments [20].

Individual differences in implicit cognition exert a profound influence on social behaviour, including attitudes, stereotypes and self-concept. Their assessment poses one of the most intriguing challenges in psychological measurement. In addition to projective testing and similar interpretive methods traditionally employed to assess ‘the unspoken’, recent developments in cognitive methodology offer a host of new methods ranging from priming [21] and implicit association [22] through semi-projective techniques [23] to performance based methods such as video-game embedded assessment protocols [24], [25].

Recently, the utility of implicit measures of social cognition have been investigated in relation to doping. A recent study [26] showed that the adapted Implicit Association Test (IAT) has the capacity to uncover automatic evaluative bias toward doping among self-confessed users and was able to predict behaviour in hypothetical situations above and beyond the explicit measures. Although the authors concluded that the doping IAT could further benefit from a refined stimuli set and improved protocol, the results indicated that implicit assessment of doping attitude has the ability to make a key contribution to the understanding of cognitive processes behind doping behaviour. A study using an emotional Stroop task with doping words suggested that allocation of attentional resources presents among young adolescents, but the source of this attentional bias has remained speculative [27]. Young people might be tuned for doping related stimuli because of external exposure (media, anti-doping education), and not necessarily internal motivation.

### Assessment of doping attitude-behaviour links

The majority of the quantitative research into doping behaviour has been based on self-reports, where athletes are not only asked to report on their own attitudes, perceived injunctive and/or descriptive norms but also asked to confess their compromising behaviour (i.e. taking prohibited substances). Self-reports among athletes in Olympic sports have yielded prevalence data ranging between 1 and 30%, which itself is higher than the yearly rate (∼2.%) of adverse analytical findings in the World Anti-Doping Agency accredited laboratories [28]. This 2% constitutes a yearly average of some 3,500 positive tests.

### Alternative approaches to self-report methods

Despite the widespread use, self-report techniques come with considerable limitations. With regard to self-reported behaviour, it must be assumed that individuals are willing to disclose this, often discriminating, information to the researcher. When self-reports are used to assess social cognitive processes, it is further assumed that people have introspective access to the construct in question (e.g. attitude) and have no intention of distorting their responses. Violations of either of these two assumptions negate the validity of self-report assessment and conclusions derived solely from self-declared data.

Doping is a decidedly ostracised behaviour. Admitting use or even expressing supportive opinions against the general view is likely to prompt many athletes to conceal their true behaviour and thoughts about doping if they could be discriminating for the person or the group he/she represents. Recently, researchers have recognised this problem and made attempts to use indirect methods to obtain information on doping behaviour. One notable example being the use of the Random Response Technique (RRT) where estimation of doping prevalence is made on aggregated levels [29], [30]. Another line of research has made attempts to estimate the likelihood of self-involvement in doping utilising the False Consensus Effect (FCE) which has been evidenced in various socially sensitive situations [31], [32]. Despite the advances these latter approaches have brought to doping behaviour research, results still carry the inevitable caveat of being based on self-declarations. Independent validation or calibration [33] of these results remains an issue.

### Objective verification of self-reported drug use

Previously reported validity studies of self-reported drug behaviour used chemical analysis for the presence of mainly social drugs in urine, saliva or hair [34]–[39]. Beyond the expected discrepancies, it was also demonstrated that inconsistencies in self-reported drug use by adolescents are not random but are associated with socioeconomic parameters, personality characteristics and/or underlying social cognitive determinants [35]. For example, reporting and under-reporting of drug use was discordant and driven by social desirability concerns [36]. Discordance between self-reports and objective validation also occurred in the unexpected direction with a considerable proportion (34%) of self-report data unconfirmed by urinalysis [39]. This may be explained by the difference between the time and/or duration of use, drug half-life and the detection window of the chosen chemical validation. To our knowledge, no research has been published that focuses on verifying self-reported performance enhancing drug use with chemical analysis of hair samples which covers prolonged periods.

In spite of the limited validity of self-reports in socially sensitive behaviour being well documented, how this discrepancy affects the conclusions drawn on the differences in social cognitive measures between those involved vs. those who are abstinent remains unknown. Whereas social psychology research routinely considers the effect of social desirability on explicitly assessed data, we are unaware of studies that investigated differences in related social cognition under different scenarios where user vs. non-user groups were established based on self-report admissions, chemical findings or validated self-reports, and used both explicit and implicit assessments. Therefore, the aims of this study were: i) to contrast self-reported doping use with objective results from chemical hair analysis and ii) to investigate the influence of the discrepancy on doping attitudes, social projection, descriptive norms and perceived pressure to use doping.

### Aims

Previous research using a larger sample pool, from which the current study sample was selected, investigated the FCE regarding doping and social drug use and provided compelling evidence of the differences in projected use of doping among peers and attitude between those athletes who confessed to having personal experience with doping and those who claimed no use [32]. The differences were in the expected direction with self-confessed doping users giving higher prevalence estimates, showing a more lenient explicit attitude toward performance enhancements than their no-user counterparts. In this study, we expanded the investigation by using hair analysis to verify self-reported doping use or abstinence, and added implicit assessments in a selected group of athletes.

We hypothesised that:

H1: Accurately reported doping use was expected to be associated with more positive explicit doping attitudes and higher estimates of projected use by others; while denied use would lead to lower explicit attitudes but realistic or elevated estimates of doping use in others; and accurately reported abstinence (‘clean athletes’) would be associated with relatively low scores on both measures.
H2: Doping use was expected to result in greater correlation between explicit and implicit doping attitudes, whereas larger discrepancy between explicit (cognitively controlled) and implicit (‘unconscious’) measures was expected in those who do not use doping.

## Results and Discussion

### Verifying self-reported doping behaviour

Hair samples from the participants in our previous study [32] were tested for performance enhancing and social drugs. Of the 82 athletes, 12 (14.6%) reported having personal experience with prohibited performance enhancing substances, one with therapeutic use exemption. Twelve hair samples were positive for anabolic steroids and/or erythropoietin (EPO), of which 10 (12.2%) were confirmed with no overlap between confessed lifetime experience and current use. None of the positives reported medical use of anabolic steroids or EPO. The pattern was very similar for social drugs with 15% overlap between self-reported use (27, 32.5%) and current use (12, 14.6%). Three of the confirmed doping positives also tested positive for social drugs.

The observed discrepancies between self-reports and objectively verified social drug taking behaviour is in line with previous research and although not surprising, they highlight the fact that a significant proportion of respondents simply choose to deny their real current or recent behaviour, even under circumstances when the verification is known to the participants. This phenomenon that has already cast doubt over drug use survey research expands to, or even magnifies the unreliability of doping use epidemiology surveys. The evaluation of anti-doping interventions is seriously hindered by the absence of reliable information on athletes' true behaviour; opens the field to wild guesses and speculations, often about other athletes, sports and nations. Devising more reliable ways to gauge this crucial information is an important issue but beyond the foci of this research and shall be addressed by future research. The present investigation aims to interpolate the tendency of giving misleading information about the behaviour to selected self-reported social cognitive processes.

Hair sample results were combined with self-reported doping use to inform the selection of 14 athletes to populate the groups in **Table 1**. Among the athletes selected for this study, 4 athletes admitted having used performance enhancing substances (PEDs) with no (or undetectable) current use. Of the remaining 10 athletes claiming that they have never used such substance 6 hair samples were positive for steroids, with all samples but one showed above the level for stanozolol, and one for nandrolone. Of these 6 athletes, 2 tested positive for a selection of social drugs despite that they both denied such drug use.

**Table 1:** Mean tests results (±SD) for self-report measures and implicit association effects (implicit doping attitude) by user groups.

Declared group membership	Objectively confirmed group membership	Explicit doping attitude (raw scale score)	Implicit doping attitude (IAT effect, ms)	Perceived pressure to dope (raw scale score)	Social projection (raw scale score)
**Non-user**	‘Clean’	29.00±6.73	−255.98±153.46	2.50±5.00	32.50±26.30
	‘Denier’	28.50±4.93	27.48±132.41	0.00	9.50±12.50
	‘Repeat denier”	34.00±6.06	−17.91±99.13	0.00	20.00±28.28
**Self-reported non-user group:**		29.90±5.64	−94 98±185.18	1.00±3.16	20.80±22.12
**User**	‘Unconfirmed self-report’	47.25±6.90	−140.56±129.85	37.50±37.97	52.50±25.00

Based on self-report and hair analysis results for doping substances, athletes were categorised into disjoint groups of: i) clean athletes (matching negative self-report and hair screening), ii) denier (negative self-report coupled with positive hair samples), iii) open users (matching positive self-report and hair) and iv) unverified/non-current user (admitted use with currently negative hair sample). Although hair samples were also tested for recreational drugs (5 out of the 10 positive samples for doping were also positive for recreational drugs), parallel psychological testing was only performed in relation to doping, hence the confessed use of recreational drugs and/or positive hair samples for such substances will not be addressed in this report. In our previous study we have shown that whilst self-reported use of recreational drugs and doping substances was not independent, related social cognition were domain specific [32]. That is, self-admitted doping users gave significantly higher estimates of doping prevalence among athletes but not social drug prevalence, and vice versa. Similarly, differences in doping attitude scores were related to doping use but independent of social drug use. However, two athletes in the current sample denied any type of drug use whilst their hair samples contained evidence of both PEDs and social drugs. As this category of athletes demonstrated repeated denial on a single survey, they were treated as a separate group in this study.

### Attitudes, perceived pressure and social projection by user groups

**Figure 1:**
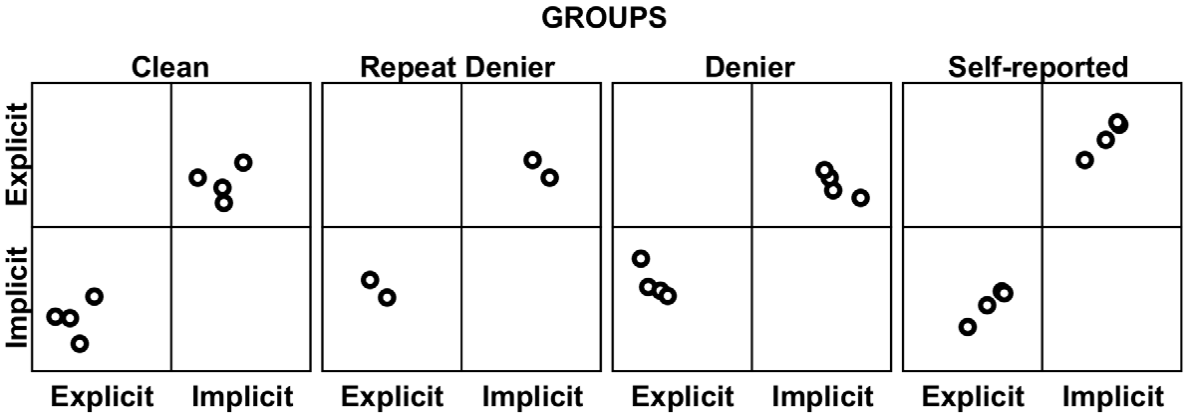
Scatterplot between explicit doping attitude scores (‘explicit’) as measured by PEAS and implicit doping associations (‘Implicit’) as measured by the Brief IAT-D by doping user groups.

Assuming that direct experience increases attitude salience and the level of attitude - behaviour consistency [40], athletes' explicit and implicit attitudes and social projections were contrasted in the four user groups. The relationship between explicit and implicit doping attitude was investigated separately in each group (with repeat deniers excluded from the analysis owing to the insufficient variation in the sample) but included in **Table 1** and **Figure 1**.

Prior to in-depth analysis, it is important to note the distinction between the two types of information. In the questionnaire phase, participants were asked if they have ever used performance enhancing substances or social drugs. Hence an affirmative to this question does not necessary mean current or recent use. Hair analysis covered approximately the last 3 months (minus the last 2 weeks when the hair is still in the body); therefore results reflect relatively recent use. It should be noted that the hair analysis at this stage was limited to the list of most often used performance enhancing and social drugs. Contradicting answers can be derived from two legitimate sources: i) respondent answered truthfully about having an experience but the last occasion when drugs were used happened before the 6-month maximum detection window; or ii) the drugs used were not among those tested for. Theoretically there is also a possibility that a respondent did not answer truthfully but there is very little reason to admit a socially unacceptable behaviour when it in fact did not happen. On the contrary, a ‘no’ answer on the questionnaire coinciding with positive analytical results in the matching hair sample can be seen as a denial on the self-report because the denied ‘ever use’ is contradicted by the very presence of a drug or drugs in the hair.

#### Explicit measures

In self-reports, ‘deniers’ and ‘repeat deniers’ are classified as ‘non-users’ with explicit scores and measures below those who admit to doping and close to those who are truly clean. This phenomenon holds clearly for two of the three explicit measures, doping attitude and perceived pressures. Interestingly, social projections were given the lowest percentage by those who denied doping use where hair results indicated otherwise and reached the highest estimation by those who admit using PEDs. Users denying their actions claimed that they feel no pressure at all to use PEDs, followed by the clean athletes (with a low 2.5%) and self-admitted users scoring the highest with 37.5%. Correspondingly, 3 out of 4 of the self-admitted users believed that most high-performing athletes used performance enhancing substances in training and competition with the 4^th^ athlete believing that doping is used by most athletes in training but not in competition. Of those who denied doping use but their hair samples indicated otherwise, half (3/6) agreed that performance enhancing substances are used in both training and competition by most high performing athletes, followed by 2/6 stating that most athletes do not use doping (1 in each ‘denier’ group) with 1 athlete believing that doping is used by most high performing athletes but used only in competition. This view was generally shared by the clean athletes, where 2 of the 4 thought that doping is used in both training and competition with the remaining 2 votes being split between training only and competition only.

Therefore, relying solely on self-report data, the observed differences in deliberate judgment were in the direction expected from known groups, with differences in three of the four measures reaching statistical significance. These are, in diminishing order of significance: explicit attitude (|t| = 4.901), pressure to use PEDs (|t| = 3.217) and social projection (|t| = 2.343; all *t*s<CV = 1.782 directed, at df = 12, α = 0.05). In reality, however, the membership of the self-reported non-user group was seriously confounded by a number of distorted answers about athletes' doping use. When these denials were corrected by hair analysis verification, a considerably different pattern of group differences emerged.

The highest estimation of doping prevalence given by self-confessed users is consistent with previous results [31], [32]. The elevated estimation may be explained by the desire to find comfort in big numbers (also called False Consensus Effect) by which people who are involved in a socially disputable act tend to overestimate the number of others doing the same [41]. The opposite trend has also had some support from literature [42], when socially endorsed behaviour may correlate with slight underestimation of the proportion of other well-behaved individuals to reinforce one's uniqueness. Our results are consistent with this observation: our self-reported non-users, overall, gave a considerably lower estimation (21% vs. 52%) of doping prevalence in others. However, past research has mainly based these interpretations on self-reported behaviour. With the added insight from the hair analysis, the description of this phenomenon can further be refined. Contrary to the expectation, those athletes who denied PED use did not give realistic or elevated estimates of doping use in others. In fact, their projection was the lowest among all groups. Those who were determined to create a good impression to hide their real behaviour gave a very low estimate of doping prevalence, scored the lowest on the explicit doping attitude scale (indicating strong disapproval) and claimed that they felt no pressure at all to use PEDs. By contrast, they performed the implicit doping association task with ease when doping words were combined with good words; the task that was more difficult to non users, for whom doping had little or no salience. As would be expected, self-admitted users' performance on the same task fell somewhere in between. All together, the discrepancy of the different inferences that could be drawn under the two scenarios (self-report vs. validated behavioural data) highlights not only the unreliability of self-reports in social sensitive domains but also their effects on related constructs of social cognition.

Theoretically there are two fundamental and mutually exclusive assumptions underpinning the observed low scores on explicit social cognition measures among verified doping users. On the one hand, it may be reduced introspective accessibility of the constructs in question: having no insight into their feelings and biases the respondents produced low scores are no reflection of their actual doping-related cognition, but instead represent an extraneous influence, such as generic social desirability. On the other hand, answers on the explicit tests are consciously and deliberately distorted in order to create a favourable (but false) impression. Our results, however, suggest that objectively verified doping users had, in fact not only introspective access to the construct (doping attitude) but also had positive feelings toward it. Investigating accessibility effects on varied implicit social cognition, Gawronski and Bodenhausen demonstrated that performance on a latency-based response compatibility task (such as the IAT) is affected by the practiced ease of and subjective feelings about the retrieval of relevant information (i.e. valence attached to doping) from memory [43].

#### Implicit measures

By contrast, the implicit association test was more revealing. Responding to pairings of positive-connotation words (the ‘good’ category) with doping substance words was fastest among those who currently use doping but denied it, followed by those who are currently using doping and admit it. Not surprisingly, responding to the same word pairings was slowest for those who claimed to have no experience with doping, followed by those who reported having used PEDs. Interestingly, athletes with doping experience performed the task quite well, indicating a closer association of doping with positive connotations than observed in those who have not used doping. Current users, as indicated by their hair analysis results, performed the *good+doping* pair the fastest with the results being close to the goo*d+nutritional supplement* pairing. These differences, however, are very small with large variance, based on small groups, and hence should be treated as preliminary observations, rather than definite conclusions.

### Relationship between explicit and implicit measures: indicators for method development

The triangulation of self-reported explicit measures and objective verification of behaviour data using hair analysis with an implicit measure provided some preliminary evidence that the reason behind underreporting explicit cognitions is not a genuine effect but more likely a strategic response. In order to take a step forward to identifying deniers without the advantage of hair sample analysis, we examined the correlation between explicitly and implicitly assessed doping attitudes separately for the four groups. In the literature, the correlation between explicit and implicit measures of the same construct tends to be small [44]. This is especially true when social desirability is thought to confound explicit responses. In several studies, implicit measures had incremental predictive power in criterion validity over and above self-reports in socially sensitive domains [45]. Scatterplots by user groups depicted in **Figure 1** and corresponding correlation coefficients in **Table 2** suggest that the relationship between the parallel explicit and implicit measures is indicative of deliberate distortion. We assume that the implicit association is close to the true reflection of people's feelings toward the attitude object. For example, those who endorse doping would be able to perform the lexical sorting task of doping words when they share the same key with positive-connotation words faster compared to those who associate doping with negative connotations.

**Table 2:** Correlation between explicit and implicit doping attitudes by user groups.

	PEAS * Brief IAT-D (average time diff)	PEAS * Brief IAT-D(d-score)
**‘Confirmed clean’ (n = 4)**	.281	.270
**‘Self-reported user’ (n = 4)**	.991	.942
**‘Denier’ (n = 4)**	−.868	−.951

Athletes who honestly admitted PED use performed congruently on the explicit and implicit measures. The more they endorsed doping in self-report and deliberate judgement, the faster they performed the *good+doping* pair test. Note that this is a trend between the two measures. In terms of sign of their attitudes, even these athletes were negative towards doping, albeit not as negative as their non-user counterparts.

Interestingly, trends expected and observed in research using self-reports change dramatically when the behavioural categories are based on objective measures (chemical analysis) and not on self-reports. Whilst patterns of explicitly assessed social cognition and tend to be consistent with self-reported behaviour, data from hair analysis revealed that distorted responses tend to bias these results. It can be argued that cultural context (i.e. doping use is unaccepted, un-sportsmanlike behaviour) influenced the athletes' automatic associations when performing the IAT task, as evidenced by the general trend of doing better on the *good+nutritional supplements* pair compared to the task when the *good+doping* shared the same response key [44]. If that is the case, athletes who denied PEDs use appeared to be less affected by this, showing little differences in response latencies between these two tasks.

The fact that self-reports on behaviour are very consistently associated with explicit social-cognitive outcomes is indeed informative. It is also consistent with mainstream social cognition literature [44]–[46] linking self-report to consciously controlled, deliberate outcomes – as distinct from more automatic and less controlled outcomes linked to implicit attitudes and dispositions. In our context this could indicate how athletes want to be seen to the outside word. In future studies, the strength and effect of this desire should be taken into account in explicitly measured doping-related constructs. Self-reports reflect what respondents want to reveal about themselves in that particular context, which has a non-trivial relationship to their actual feelings, thoughts or behaviour.

### Limitations

Following the recommendation [44], we use the terms explicit and implicit with reference to measurement, not the construct (e.g. attitude). Based on the implicit doping attitude data at hand, no assertions can be made about the level of awareness among the selected athletes, especially in the denier group, of their own attitude demonstrated in the IAT. Rather, the considerable discrepancy between explicitly and implicitly measured attitudes in the denier group only differ qualitatively in their doping behaviour and their willingness to disclose this information suggest that athletes, indeed, were aware of their attitudes but owing to the sensitive nature of the issue, they made a deliberate effort to conceal their feelings about doping when it was under their cognitive control (i.e. explicitly measured) and deliberated. By contrast, automatic activation of these attitudes during the implicit association test was something that is very difficult to manipulate at will. The fact that the task was presented as a timed exercise to respondents who were competitive athletes may have further enhanced the validity of the test. That is, athletes were likely to be focused on performing fast and accurately on the task, instead of pondering about what the test might be measuring.

Limitations of this study arise from the sample size. Whilst the number of hair samples screened and positives samples confirmed are considerably higher than what is typically used in publications focusing on the chemical analyses for steroids [47], [48], it is somewhat below the typical sample size in similar experimental psychology studies [45]. Results from this study were presented as evidence for the need for chemical validation of self-reports and mixed methodology, rather than drawing firm inferences regarding user vs. non user groups. In order to do this, similar investigations need to be conducted on sufficiently large samples to establish representative groups and improve confidence with which practically meaningful differences/relationships can be observed. All together, the discrepancy of the different inferences that could be drawn under the two scenarios (self-report vs. validated behavioural data) highlights not only the questionable validity of self-reports in social sensitive domain but also their profound effects on related social cognitive outcomes. Sample descriptions (e.g. means and standard deviations) in this study are only indicative and presented here to assist in estimating the required sample sizes for future studies.

### Conclusion

Incorporating developments in hair sample analysis for the detection of performance enhancing substances, this initial study examines the prospects of objective validation of athletes' doping attitude estimations and admissions of use. Overall the results indicate that patterns of group differences in deliberately expressed attitudinal outcomes, such as social projection, explicit attitude to doping and perceived pressure to use, vary depending on whether the user and non-user groups are defined by self-report or by objective verification such as hair sample analysis. When user and non-user groups were defined by self-report, the differences between them on several attitudinal outcomes were observed in the expected direction (i.e. self confessed user groups scored higher on social projection, explicit attitude to doping and perceived pressure to use). However, data from hair analysis revealed that deliberate response distortion may have biased these results. Subjects, whose hair sample returned positive for doping but who denied doping use in self-reports, were observed to manipulate their questionnaire responses to a greater degree than all other groups. Implicit doping attitude and its correlation to the explicit attitude towards doping are indicative of this distorted responding.

Therefore, the observed discrepancy between self-report and objectively (e.g. chemically) validated behavioural data needs to be considered when drawing conclusions from self-report findings. Our results pose a challenging question about the veracity of studies where doping-related behaviours and attitudinal outcomes are examined through group or individual differences that are themselves based on self-report. Our findings not only confirm the need for improved self-report methodology for future research in socially-sensitive domains but also indicate where the improvements are likely to come from: as chemical validation remains expensive, a more realistic promise for large scale studies and online data collection efforts is held by measures of implicit social cognition.

Owing to the time and resource-intensive nature of chemical validation (including equipment, personnel and know-how), large scale adoption of such validation for self-reported behaviour data across doping research does not seem feasible. However, improving self-report methodology remains imperative. One possible avenue is incorporating implicit assessments to gain incremental predictive validity over and above explicit self-report measures. This approach has also been advocated by Greenwald et al. [45] upon meta-analysis of 122 empirical studies using explicit and implicit measures to predict behavioural, judgemental and physiological outcomes.

## Methods

The chemical validation of self-reported information on doping and drug taking behaviour was part of a multi-centre study investigating social projection in doping and social drug use [31], [32]. This part of the study aimed to detect the presence of selected drugs and metabolites in hair in order to investigate the validity of self-reports and the effect on any expected discrepancy between self-declaration and objective behavioral data on doping related social cognition.

### Design

**Figure 2:**
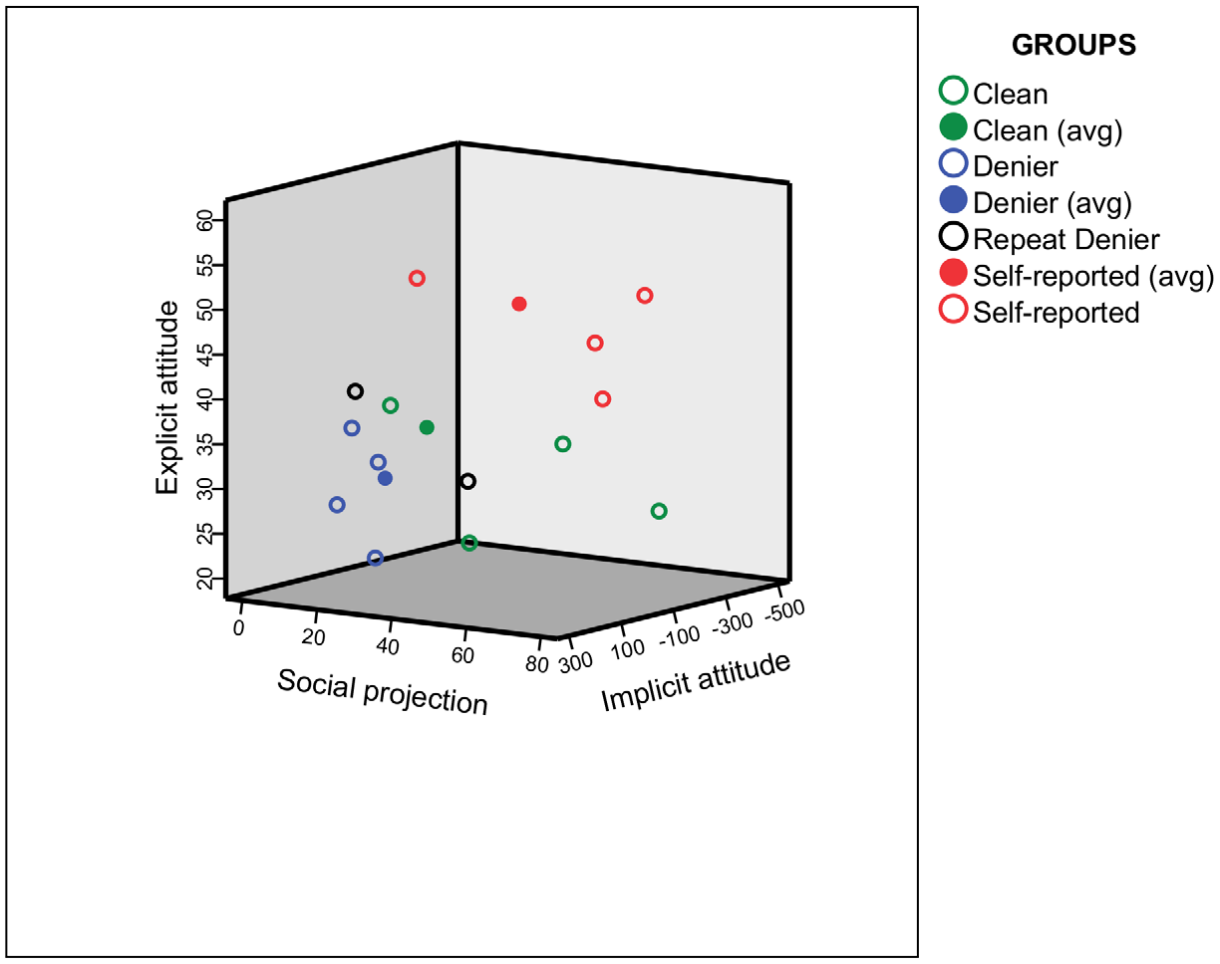
Sample characteristics and group means for 2 explicit and 1 implicit assessments.

This study was based on mixed methods using a questionnaire, computerised psychological test and hair analysis for selected performance enhancing drugs. Self-report questionnaire results on doping behaviour were compared to the data gleaned from hair sample analyses for 14 selected athletes (4 per group plus 2) based on their self-reported behavior and hair sample results from the ELISA screening. In groups with more than 4 athletes (e.g. ‘clean’, ‘denier’ and ‘self-reported’), 4 athletes were randomly selected for confirmation and further testing. The sample pools were as follows: 61/115 clean athletes, 11/115 self-reported users (only 1 was confirmed), and 12/115 deniers (2 erythropoietin and 9 steroids users, one was not confirmed). The representativeness of this random selection is shown in **Figure 2**. Participants with unconfirmed positive ELISA results were eliminated from the sample pool. Participation was anonymous, voluntary and based on fully informed written consent. Participants were told that the hair samples will be analysed for various chemicals. All athletes were aware of the hair sampling procedure before completed the questionnaire and performed the computerised assessment. As the completion of the testing protocol required at least one hour, participants were compensated for their time with a small payment (value of less than 10 Euros).

### Ethics statement

The study was approved by the Faculty Research Ethics Committee in Kingston University.

### Procedure

Athletes were asked to complete a web browser based test consisting of the explicit and implicit attitude measures, complemented with a paper-and-pencil questionnaire. A brief self-esteem IAT with ‘good’, ‘bad’, ‘self’ or ‘others’ stimuli set separated the two doping measures and served as method practice. Results for the implicit self-esteem test are not reported in this study. Implicit assessments preceded the explicit questionnaire measures (including questions about PED use), separated by other, non-doping related computerised tests, hence explicit did not influence the implicit assessment [44]. Although respondents were presented with an Information Sheet detailing the hair sampling procedure when seeking consent, the emphasis of the research was not on doping but investigating resource depletion in executive functioning, where doping appeared to be one avenue of evoking self-control and was mixed with other tasks (e.g. Donders' task switching and Stroop response inhibition).

Testing took place in a well-lit, quiet room containing two desktop computers. One or two athletes were present and completed the task at a time under supervision. The data collection was conducted between 8 am and 6 pm during weekdays.

### Validation of self-reports

Validation of self-report was conducted using hair samples. The key advantage of using hair, as opposed to blood, urine or saliva, is its wide detection window, coupled with being non-invasive, easily stored and free of biohazards. The selection of drugs for screening was based on frequency of detection in WADA reports over the past five years [28]. Thus, along with testosterone, stanozolol, nandrolone and boldenone are frequently used anabolic steroids which differ in their licensing status [49]. In addition, tests were conducted for Naltrexone and most commonly used recreational drugs (for the full list, see **Table 3**). This research has adhered to the WADA CODE for laboratories [50]. Proper chain of custody was followed for hair samples collection, storage and disposal. Any unusual conditions like colour, pH and specific gravity were recorded.

**Table 3:** Limits of detection (LOD) and WADA general Minimum Required Performance Limit (MRPL) values.

Drugs	Category	ELISA LOD	MRPL value
Nandrolone	Anabolic steroid	0.07 ng/ml	2 ng/ml
Testosterone	Anabolic steroid	0.5 ng/ml	2 ng/ml
Naltrexone	Anabolic steroid	1.3 ng/ml	2 ng/ml
Boldenone	Anabolic steroid	6 ng/ml	2 ng/ml
Stanozolol	Anabolic steroid	1 ng/ml	2 ng/ml
3-HydroxyStanozolol	Anabolic steroid	12 ng/ml	2 ng/ml
Amphetamine	Stimulant	11.5 ng/ml	500 ng/ml
N-Desmethylselegiline	Stimulant	1.27 ng/ml	500 ng/ml
Ephedrine	Stimulant	23.4 ng/ml	500 ng/ml
Methamphetamine	Stimulant	9.5 ng/ml	500 ng/ml
Δ^8^ THC	Stimulant	0.6 ng/ml	500 ng/ml
Δ^9^ THC	Stimulant	0.5 ng/ml	500 ng/ml
Cocaine	Stimulant	5.1 ng/ml	500 ng/ml
Cocaethylene	Stimulant	5.5 ng/ml	500 ng/ml
Benzoylecgonine	Stimulant	6.8 ng/ml	500 ng/ml
m-Hydroxycocaine	Stimulant	7.1 ng/ml	500 ng/ml
Ketamine	Stimulant	7 ng/ml	500 ng/ml
Norketamine	Stimulant	137 ng/ml	500 ng/ml
EPO	Peptide hormone	1.2 mU/ml	5 mU/ml or 40 pg/ml

### Hair samples

The hair sample consisted of a lock of untreated hair with a diameter of 3 to 4 mms (approximately 50 hairs), minimum 3 cm in length (equal 100 mg in weight), cut directly at the skin surface at the vertex posterior whenever possible. The sample was stored individually in labelled, sealable paper envelopes, according to the protocols established and approved by the Kingston University Faculty Research Ethics Committee.

#### Chemicals and reagents

ELISA kits for nandrolone, stanozolol, amphetamine, methamphetamine, cocaine, delta-9-tetrahydrocannabinol (THC), ketamine, erythropoietin (EPO), and their metabolites, were obtained from Neogen Corporation (Lexington KY 40511 USA), with enzyme immunoassay (EIA) being part of the ELISA kits. Drugs, their metabolites and internal standard (stanozolol D3) were obtained from LGC standards (Teddington, UK). All chemicals and silanized amber glassware were from Sigma Aldrich (UK). Blank hair was obtained from healthy non-athlete volunteers.

### Screening by ELISA

The hair sample was rinsed twice with 5 ml dichloromethane for 2 minutes. After complete drying, hair was finely cut into circa 1 mm segments. Hair segments (ca 50 mg) were weighed in a glass tube. Calibrants and controls for each kit were prepared by spiking blank hair with the required amount of drug. Hair samples, calibrants and controls were then incubated in 1 mL of 1 M NaOH at 95°C for 15 minutes. After cooling, the homogenate was neutralized (pH 7) with required amount of 1 M HCl (approx 1 mL) and then diluted with equal amount of enzyme immunoassay (EIA) buffer (1∶1 v/v). Screening methods were fully validated in accordance with the WADA Code of validation for urine and plasma which was extended to hair samples. Neogen Corp. (USA) forensic ELISA kits were used on a Biotek-ELx808 (USA) and Varian Cary 50 MPR Microplate Reader (UK). The full range of drugs and their metabolites are given in **Table 4**. In addition to the steroid results, the application of the Neogen ELISA methods have been extended from biofluids to hair samples for the detection of EPO and the most frequently used drugs of abuse that are on the WADA 2009 List of Prohibited Substances [51]. These include amphetamine, methamphetamine, cocaine, marijuana and ketamine (currently not prohibited) and their selected metabolites. This process involved developing extraction methods along with devising a protocol for analysis. Methods for extraction of the drugs from hair were developed and subsequent ELISA analyses were validated in-house.

**Table 4:** Limits of detection using LC-MS/MS.

Drugs	Category	MRPL value	LC-MS LOD		Calibration curve in hair pg/mg
Nandrolone	Anabolic steroid	2 ng/ml	1 ng/ml	2.5 pg/mg	3 to 400
Testosterone	Anabolic steroid	2 ng/ml	0.1 ng/ml	0.25 pg/mg	1 to 400
Stanozolol	Anabolic steroid	2 ng/ml	0.2 ng/ml	0.5 pg/mg	1 to 400

For all non-threshold and threshold substances appropriate controls near the appropriate threshold levels were included in the initial screening, although uncertainties of measurements were not taken into account. **Table 4** shows the detection limit of ELISA kits supplied by Neogen Corporation (USA) and general MRPL levels set by WADA.

### Confirmation

The ELISA results were confirmed by liquid chromatography-mass spectrometic (LC-MS/MS) methods using a ThermoScientific LC-MS/MS Accela UPLC coupled with Triple Quadrupole TSQ™ Quantum Access system. These confirmatory quantitative methods are more sensitive than the initial screening procedures with the LOD's of the three key substances in hair are shown in **Table 4**. There are no therapeutic use exemptions (TUE) for the prohibited substances detected.

#### Analyses by LC-MS/MS

After decontamination, hair was finely cut into 1 mm segments. Following a previously established method [52], hair segments (ca 20 mg) were weighed in a glass tube and incubated in 1 ml of 1 M NaOH at 95°C for 15 minutes in the presence of stanozolol D3 as an internal standard (I.S). After cooling, the homogenate was neutralized with approximately 1 mL of 1 M HCl followed by addition of 0.2 M phosphate buffer (pH 7.0). Liquid – Liquid extraction was employed for all three steroids analyzed. Pentane (3.5 ml) was added to the homogenate. After agitation and centrifugation (4 minutes at 1257 g) the organic layer was separated and evaporated to dryness under a stream of nitrogen gas at 60°C. The dried residue was reconstituted with 100 µL acetonitrile. An aliquot (4 µL) of reconstituted extract was injected into the ThermoScientific LC-MS/MS system.

#### LC-MS/MS conditions

An Agilent ZORBAX column (SB-C18, 2.1×50 mm, 1.8 µm) was used. Formic acid (0.1%) and acetonitrile were used as mobile phase. The LC mobile phase gradient flow used was: A: acetonitrile (%), B: 0.1% formic acid; start: 50% A, after 10 min: 80% A–20%B, after 11 min: 100%A, after 12 min: 50%A. Total flow rate through the column was set at 100 µl/min using gradient flow. Column temperature was set at 60°C. The mass spectrometer was operated in the positive electrospray ionisation mode. SRM (single reaction monitoring) was used to confirm each analyte as shown in **Table 5**. A standard calibration curve and quality controls were prepared by spiking negative control of hair (blank hair) with the required amount of drug and internal standard.

**Table 5:** Main qualifier ions of analytes used for steroid analysis.

Analyte	Parent mass	Product mass
Nandrolone	275.2	109.2
Stanozolol	329.2	81.2
Testosterone	289.2	109.2, 97.2
Stanozolol D3 (I.S)	332.2	81.2

### Psychological assessments

Psychological assessment consisted of computerized word sorting task (used to assess implicit associations) and a paper-and-pencil questionnaire seeking information on explicit doping attitude, and basic demographic information (gender, age, ethnicity, sport, level of competition, nationality). In order to protect athletes' anonymity, only mean age and gender distribution is reported.

#### Implicit doping attitude (the brief version [53])

In this test block, respondents were presented by words falling into four categories (good, bad, nutritional supplements or doping). The stimuli used in each category are shown in **Table 6**. Two of those four category names were shown on the left hand side of the screen during the test. Respondents were asked to press ‘E’ if the stimulus word matches either of the categories or to press ‘I’ if it does not match them. Words were presented in 24pt Arial font. Each stimulus was preceded by a fixation cross which stayed on-screen for 400 ms. Stimuli stayed on-screen until the respondent pressed either ‘E’ or ‘I’. A large red X was shown on the bottom of the screen for 400 ms when the answer was wrong; respondents had to press the correct button to proceed.

**Table 6:** Stimuli of the Brief Implicit Doping Attitude test.

Category	Words
Good	peace, joy, love, smile
Bad	sick, hell, poison, fail
Doping	nandrolone, stanozolol, testosterone, amphetamine
Supplements	vitamins, ginseng, garlic, calcium

The Brief Doping IAT test consisted of two blocks. In the first block, categories ‘good’ and ‘nutritional supplement’ were assigned to the ‘E’ key; the second block used categories ‘good’ and ‘doping’. Each block consisted of 32 stimuli and each word was presented twice. Brief instructions were presented before each block; the instructions specified the words of the categories that were selected as target categories (i.e. good and nutritional supplement in the first block; good and others in the second block) but not the other two. The ‘good’ combinations (good + nutritional supplement and good + doping) were fixed as focal categories. Respondents were instructed to proceed as fast as they could. The order of the two blocks was counterbalanced.

**Figure 3:**
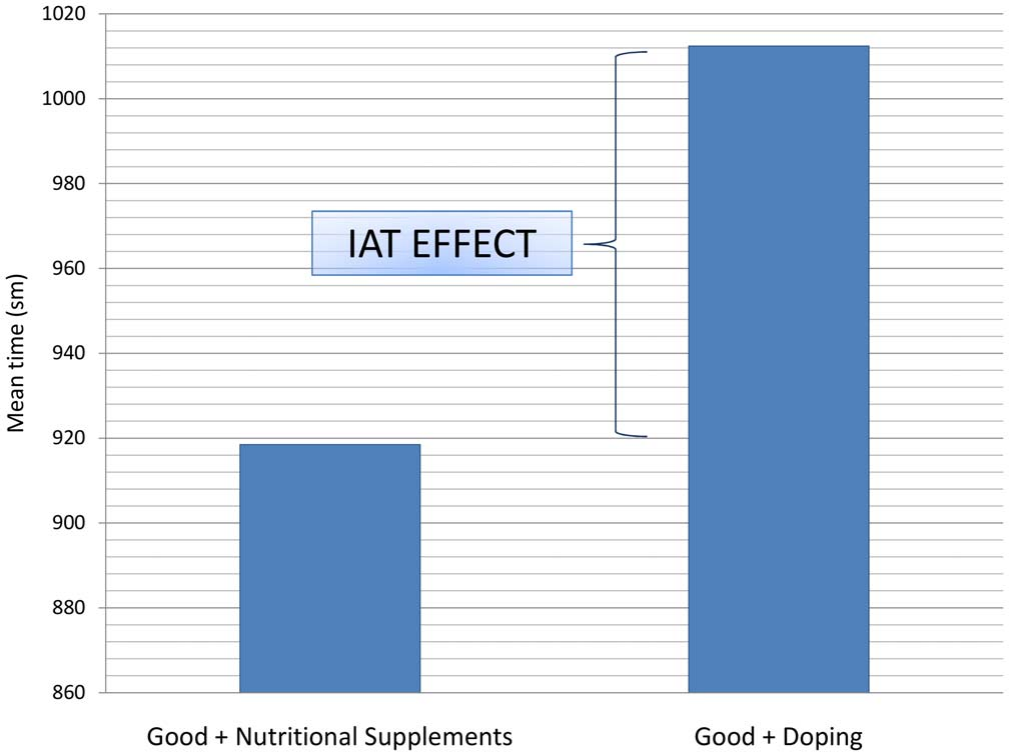
Illustration of the Implicit Association Test (IAT) effect.

The Doping IAT effect was calculated as the difference time difference between the two focal test blocks as shown in **Figure 3**. The difference was also divided by the variance to derive the D-scores [54]. Because the difference was calculated as: [*Good + Nutritional Supplement*] – [*Good + Doping*], difference time>0 means that completion of the good + nutritional supplement combination task took longer, whereas difference time<0 suggests that the [*Good + Doping*] completion took longer.

The computerised test application also included an explicit measure of doping attitude using the Performance Enhancement Attitude Scale (PEAS). The PEAS consists of 17 statements related to performance-enhancing drugs. Respondents were asked whether they agree with the statements. Answers were recorded using a 6-point Likert-type scale (1 = strongly disagree, 6 = strongly agree). The PEAS has shown good evidence for scale reliability and validity [55].

The anonymous questionnaire included key questions on drug and doping taking behaviour: *Have you ever used a social drug*? (Yes/No); *Have you ever used a banned substance*? (Yes/No) and *Do you use nutritional supplements*? (Yes/No). The question regarding nutritional supplement use (beyond and above the normal diet and taken in a concentrated form) was included as a control (not reported). At the beginning of the questionnaire, athletes were presented with clear definitions: ‘doping’ or ‘banned substances’ were those substances that are prohibited by the World Anti-Doping Agency or other governing body in training and/or competition (e.g. steroids, EPO). ‘Social’ or ‘recreational’ drugs were defined as psychoactive drugs (e.g. stimulants, opiates, cannabis, cocaine, etc.) used for recreational purposes rather than for work, medical or spiritual reasons with caffeine, alcohol and tobacco excluded. Nutritional supplements were vitamins, minerals, and non-vitamin non-mineral substances including herbals and botanicals. Exemplars were given for all three groups. In addition to these key questions, athletes were also asked about the perceived pressure to use doping (0–100%), estimated prevalence of doping among fellow athletes (0–100%) and their general belief about the doping use pattern (descriptive norm). For the exact wording and answer options of these questions, see **File S1**.

### Sample characteristics

The hair samples of the selected 14 athletes were analysed for PEDs. Athletes competed in track & field (5), triathlon (4), volleyball (2), orienteering (1), basketball (1) and karate (1). The mean age was 20.43±3.18 years, 10 females and 4 males in the sample. In this small sample, age and gender appear to be unrelated to doping use.

**Table 7** summarised the self-report and hair analysis results for the selected 14 athletes. Note that positive hair samples for social drugs were not confirmed beyond the ELISA screening at this stage. The focus of the paper was performance enhancing drugs and social cognition relating doping, hence the test did not contain explicit or implicit measures of social cognition about social drugs. **Figure 2** shows the selected athletes' position in relation to the group mean for the full sample (N = 482).

**Table 7:** Cross-tabulated data of self-reports and parallel hair analysis (n = 14).

	Self-report: never used doping		Concentration (pg/mg)
**Hair analysis negative for doping**	**‘clean’ (n = 4)**		
	A1	–	-VE
	A2	–	-VE
	A3	–	-VE
	A4	–	-VE
**Hair analysis positive for doping**	**‘denier’ (n = 4)**		-VE
	A5	*Stanozolol, 3′OH Stanozolol*	12.65 (Stanozolol)
	A6	*Stanozolol, 3′OH Stanozolol*	11.24 (Stanozolol)
	A7	*Stanozolol, 3′OH Stanozolol*	40.24 (Stanozolol)
	A8	*Stanozolol, 3′OH Stanozolol*	56.08 (Stanozolol)
**Hair analysis positive for doping and social drugs**	**‘repeat denier’ (n = 2)**		
	A9	*Stanozolol, 3′OH Stanozolol, Amphetamine, Methampetamines,N-Desmethylselegiline, Ephedrine, MDMA, MDA,* Δ^8^ THC *THC,* Δ^9^ THC	9.82 (Stanozolol)
	A10	*Nandrolone, Boldenone,Testosterone, Naltrexone, Amphetamine, Methampetamines,N-Desmethylselegiline, Ephedrine, MDMA, MDA,* Δ^8^ THC, Δ^9^ THC	14.04 (Nandrolone)-VE (Testosterone)
	**Self-report: admit using doping**		**ELISA**
**Hair analysis negative for doping**	**‘self-reported’ (n = 4)**		
	A11	–	ND
	A12	–	ND
	A13	–	ND
	A14	–	ND

-VE = negative results, ND = - not detectable.

### Analyses

Group differences in and relationship between explicit doping attitude and implicit doping associations and social projection were compared for groups based on self-reports and hair analyses. Group means are reported with standard deviation. Independent samples t-tests were used to compare scores achieved on social cognitive measures, where user vs. non-user groups were formed by the self-reported PEDs taking. Graphs and statistical analysis were conducted by SPSS 17.0 and Excel 2007.

## Supporting Information

File S1 This file contains the questionnaire used to collect data regarding athletes' drug and doping behaviour, doping attitude, descriptive norm, social projection and perceived pressure.
(0.06 MB DOC)
