# Supplementary material for: Virtue or Pretense? Looking behind Self-Declared Innocence in Doping
Source: PLoS One. 2010 May 5;5(5):e10457. doi: 10.1371/journal.pone.0010457 (PMC2864761; doi:10.1371/journal.pone.0010457)
Supplement: File S1 — This file contains the questionnaire used to collect data regarding athletes' drug and doping behaviour, doping attitude, descriptive norm, social projection and perceived pressure. (0.06 MB DOC) [file pone.0010457.s001.doc]

**File S1: Questionnaire used to report drug and doping behaviour; and measure explicit doping attitude, descriptive norm, social projection and perceived pressure**

Please colour in the circle with the number that best reflects your agreement.

| Strongly disagree | | Disagree | Slightly disagree | Slightly agree | Agree | Strongly agree | |
| --- | --- | --- | --- | --- | --- | --- | --- |
|  | 1 | 2 | 3 | 4 | 5 | 6 |  |

| Doping is necessary to be competitive. | ➀ | ➁ | ➂ | ➃ | ➄ | ➅ |
| --- | --- | --- | --- | --- | --- | --- |
| Doping is not cheating since everyone does it. | ➀ | ➁ | ➂ | ➃ | ➄ | ➅ |
| Athletes often lose time due to injuries and drugs can help to make up the lost time. | ➀ | ➁ | ➂ | ➃ | ➄ | ➅ |
| Only the quality of performance should matter, not the way athletes achieve it. | ➀ | ➁ | ➂ | ➃ | ➄ | ➅ |
| Athletes are pressured to take performance-enhancing drugs. | ➀ | ➁ | ➂ | ➃ | ➄ | ➅ |
| Athletes, who take recreational drugs, use them because they help them in sport situations. | ➀ | ➁ | ➂ | ➃ | ➄ | ➅ |
| Athletes should not feel guilty about breaking the rules and taking performance-enhancing drugs. | ➀ | ➁ | ➂ | ➃ | ➄ | ➅ |
| The risks related to doping are exaggerated. | ➀ | ➁ | ➂ | ➃ | ➄ | ➅ |
| Athletes have no alternative career choices, except sport. | ➀ | ➁ | ➂ | ➃ | ➄ | ➅ |
| Recreational drugs give the motivation to train and compete at the highest level. | ➀ | ➁ | ➂ | ➃ | ➄ | ➅ |
| Doping is an unavoidable part of competitive sport. | ➀ | ➁ | ➂ | ➃ | ➄ | ➅ |
| Recreational drugs help to overcome boredom during training. | ➀ | ➁ | ➂ | ➃ | ➄ | ➅ |
| There is no difference between drugs, fibreglass poles, and speedy swimsuits that are all used to enhance performance. | ➀ | ➁ | ➂ | ➃ | ➄ | ➅ |
| The media should talk less about doping. | ➀ | ➁ | ➂ | ➃ | ➄ | ➅ |
| The media blows the doping issue out of proportion. | ➀ | ➁ | ➂ | ➃ | ➄ | ➅ |
| Health problems related to rigorous training and injuries are just as bad as from doping. | ➀ | ➁ | ➂ | ➃ | ➄ | ➅ |
| Legalising performance enhancing agents would be beneficial for sports. | ➀ | ➁ | ➂ | ➃ | ➄ | ➅ |

| Do you think that most high performance athletes … | |  | Tick **✓** |
| --- | --- | --- | --- |
|  | use performance enhancing substances in training and competition | |  |
|  | use performance enhancing substances in training only | |  |
|  | use performance enhancing substances in competition only | |  |
|  | do not use performance enhancing substances | |  |

| Have you ever used a social drug? |  | Tick **✓** |
| --- | --- | --- |
|  | Yes |  |
|  | No |  |

| How much pressure do you feel to use banned substances? If zero % means no pressure at all and 100% represents maximum pressure, what is the percentage that describes the pressure you feel? |  |  |
| --- | --- | --- |

| Have you ever used a banned substance? |  | Tick **✓** |
| --- | --- | --- |
|  | Yes |  |
|  | No |  |

| What % of others in your sport has used a banned substance? |  |  |
| --- | --- | --- |
